# An Assessment of Young Adults’ Awareness and Knowledge Related to the Human Papillomavirus (HPV), Oropharyngeal Cancer, and the HPV Vaccine

**DOI:** 10.3390/cancers17030344

**Published:** 2025-01-21

**Authors:** Eric N. Davis, Philip C. Doyle

**Affiliations:** 1 Laboratory for Quality of Life and Well-Being in Oncology, Rehabilitation Sciences, Western University, London, ON N6G 1H1, Canada; 2 Otolaryngology Head and Neck Surgery, Division of Laryngology, Stanford University School of Medicine, Stanford University, Stanford, CA 94305, USA

**Keywords:** human papillomavirus, cancer, awareness, knowledge, oropharyngeal cancer

## Abstract

Background/Objectives: The human papillomavirus (HPV) is a prevalent sexually transmitted infection that is a known cause of morbidities such as genital warts and cancers of the cervix, anus, and oropharynx. Non-cervical HPV-related cancers have been a developing problem in North America, increasing in incidence by up to 225% in some instances over a span of two decades. Methods: This study investigated levels of *awareness* and *knowledge* of HPV, oropharyngeal cancer (OPC), and the HPV vaccine using a self-administered web-based survey designed specifically for this research. University students (*n* = 1005) aged 18–30 completed a 42-item questionnaire that included demographic information, awareness questions, and a series of “true/false/I don’t know” knowledge questions. Results: The data gathered revealed that participants had relatively high levels of awareness. However, many respondents had significant gaps in their knowledge of HPV, OPC, and the HPV vaccine. Collectively, the data indicate that awareness and knowledge of HPV and the value of vaccination may place younger individuals at risk for HPV-related infections. Conclusions: Although a relatively high level of awareness concerning HPV was observed, the gaps in knowledge suggest that further efforts are necessary to educate young adults. While all risk factors cannot be reduced, the present data may guide future efforts directed toward better education on HPV and related health concerns and associated risks.

## Simple Summary

The human papilloma virus, commonly known as HPV, is the most common sexually transmitted infection in the world. In fact, a majority of sexually active people are at substantial risk for acquiring an HPV infection of some type in their life. Risk factors associated with young adults are substantial given that sexual activity is common in this group. This study evaluated awareness and knowledge of HPV in a large group of young adult college students. Despite a good level of general awareness in the study population, we found significant gaps which may place this population at a greater relative risk. However, risk could be mitigated to some extent through vaccination for HPV. However, considerable variability was observed in our sample of college students. Consequently, there is a need for continued education related to HPV, its associated risks, and the value of vaccination for HPV.

## 1. Introduction

The human papillomavirus (HPV) is the most common sexually transmitted infection in the world with up to 80% of sexually active people acquiring an HPV infection of some type in their life [1,2]. At present, there are more than 190 identified strains/genotypes of HPV, over 40 of which infect the anogenital tract [3]. Currently, cervical cancer is a significant global health burden, affecting over 500,000 women each year worldwide, with HPV essentially the sole cause of this cancer [4,5,6]. As such, HPV research (epidemiology, prevention, intervention) has traditionally been studied from the perspective of cervical cancer. While this work has been vital to understanding the virus, HPV research has expanded in recent decades to consider the causative role of HPV in other non-cervical cancers, including those of the oropharynx, or what is commonly termed oropharyngeal cancer (OPC).

The majority of HPV infections occur without perceptible symptoms, and approximately 90% of infections clear spontaneously within two years [7]. When symptoms do arise, they may manifest as anogenital warts (most commonly with either HPV strains 6 or 11) or as precancerous lesions of the anogenital tract, most commonly with “high-risk” strains 16 or 18 [1,8]. Anogenital warts are typically benign and pose no serious health consequences, thus, these strains are considered “low-risk”. In contrast, HPV strains 16, 18, and to a lesser extent strains 31, 33, 45, 52, 58, and others, are considered high-risk because they are found in a majority of HPV-related cancers [1].

HPV is transmitted through skin-to-skin contact with the anogenital region, including intimate sexual contact such as oral, vaginal, or anal sex [9,10,11]. However, the typical lack of symptoms associated with HPV infection and the relative ease of transmission (i.e., skin-to-skin contact) are thought to be contributing factors to the high worldwide prevalence of HPV infection [2,12,13,14]. Many North American studies have noted that the peak prevalence of cervical HPV infection occurs in university/college aged adults (age 20–24) and steadily declines after age 25 [7,15,16,17,18].

Although a majority of HPV strains are benign, certain high-risk strains are known to cause certain cancers of the cervix, oropharynx (including base of tongue, tonsils, soft palate, and pharynx), anus, vulva, and penis. Approximately 96% of all cervical cancers [19], 35–72% of oropharyngeal cancers [20,21], 78% of vaginal cancers [22], 40% of vulvar cancers [23], 84% of anal cancers [23], and 48% of penile cancers have been reported to contain HPV DNA [24]. HPV strains 16 and 18 are the most prevalent, and it has been reported that 70% of cervical cancers, 90% of oropharyngeal cancers [20,25], and 80% of anal cancers [23] may be attributed to one of these positive varieties of these two strains [12].

Epidemiological studies on HPV have expanded to include consideration of men due to the rising incidence of non-cervical, HPV-associated cancers [10,20,26,27,28]. In a large multi-national study of men aged 18–70, the prevalence of genital HPV infection has been reported at 50% [26]. Furthermore, in a large cross-sectional study of both men and women aged 14–19 in the United States, the prevalence of oral HPV infection was found to be 6.9%, with an oral HPV prevalence much higher in men compared to women (10.1% vs. 3.6%) [9,29]. Oral HPV infection was found to be eight times higher in individuals who have been sexually active vs. those who have not. Further, oral HPV infection is strongly associated with lifetime and recent numbers of vaginal or oral sex partners, confirming the sexually transmitted nature of oral infection [9,11,30]. Data from the US presented by Chaturvedi et al. on men and women aged 18–59 indicated that HPV vaccination rates had increased from 0% to 5.8% in men and from 7.3% to 15.1% in women when compared to the period from 2006–2016 [30].

### 1.1. HPV Prevalence

In 2018 in the United States, it was estimated that approximately 23.4 million males and 19.2 million females had been diagnosed with a disease-associated HPV infection [29]. A proportionally similar number of infections has been estimated to occur each year in Canada, with the estimate that 75% of sexually active adults currently have an active HPV infection. Fortunately, a large proportion of these infections occur from low-risk strains of the virus, and the majority of all infections are self-limiting. However, the most common strain, HPV 6, is known to cause genital warts, and HPV 16, a high-risk strain found in the majority of HPV-related malignancies, is the second most prevalent strain, accounting for approximately 7% of all infections [7,8,31,32].

Persistent infections, especially with high-risk strains (i.e., HPV 16 and 18) can lead to cancers of the cervix, oropharynx, anus, vulva, penis, and others. While cervical cancer still represents a significant public health burden, other HPV-related cancers are increasing [21,33,34]. This trend is especially apparent in men, who have traditionally not been considered as being vulnerable to HPV-related cancers. Thus, increasing public awareness of these non-cervical cancers may pose a valuable opportunity for prevention.

### 1.2. HPV-Related Morbidities and Mortalities

HPV-Related Cancers. Of the almost 20 million new cancer diagnoses worldwide, approximately 5% have been attributed to HPV infection [2,35,36]. In general, HPV-positive tumors are associated with better three- and/or five-year survival outcomes compared to HPV-negative tumors of the same anatomical site [37,38,39,40,41]. Gillison et al. [42] reported that individuals with HPV-positive head and neck cancers were more likely to be younger, college educated, have higher incomes, and be more sexually active compared to individuals with HPV-negative head and neck cancers. Equally important was the fact that traditional risk factors for head and neck cancer (i.e., tobacco and heavy alcohol use) had no association with HPV-positive tumors, but they were strongly associated with HPV-negative tumors.

#### 1.2.1. Cervical Cancer

Worldwide, cervical cancer is the second most common cancer diagnosed in women. There were an estimated 660,000 new cases of cervical cancer reported in 2022 worldwide, resulting in 350,700 deaths [43] (https://www.who.int/news-room/fact-sheets/detail/cervical-cancer, (accessed on 4 October 2024). In the United States, cervical cancer rates have been declining in recent decades due to improved screening measures and the development of an HPV vaccine and the recommendation for its use [44,45,46]. However, 11,500 new cases and 4000 deaths were anticipated to occur in 2024 [43] (https://www.cdc.gov/cervical-cancer/statistics/index.html#, (accessed on 13 June 2024). A proportionately similar number of women are affected each year in Canada, with an expected 1550 new cases in 2023 and 400 deaths (https://www.partnershipagainstcancer.ca/topics/eliminating-cervical-cancer/cervical-cancer-rates/#, (accessed on 12 November 2024), a rate that is over five times greater than the number of cervical cancers.

#### 1.2.2. Anal Cancer, Penile, and Vaginal and Vulvar Cancers

Although uncommon, between 84–90% of anal cancers are attributed to HPV infection [23,47]. In the first decade of this century, the incidence of anal cancer increased at an average rate of 2.8% per year [33], with the risk for cancer elevated for females who engage in anal sex and homosexual males [47]. Approximately 48% of penile cancers are attributable to HPV [24], while approximately 70% of vaginal and 40% of vulvar cancers are attributed to HPV [23].

#### 1.2.3. Oropharyngeal Cancers

Since 1990, the incidence of HPV-positive oropharyngeal cancer (OPC) has increased by 225% in the United States [2,21,48]. Overall, the American Cancer Society estimates that there will be approximately 58,450 new cases of OPC, with 12,230 deaths in 2024 [49]. Similarly, the Canadian Cancer Society has estimated that 8100 individuals will be diagnosed with OPC in 2024, with 2100 deaths; again, this is a rate that exceeds cervical cancer by five times [50]. When segmented by gender, 5800 cases will be diagnosed in Canadian men and 2300 in women; this will account for 1550 and 580 projected deaths for men and women, respectively. Overall, the proportion of OPCs identified as HPV-positive rose from 16.3% in 1984 to 72.7% between 2000 and 2004 [21], with similar trends observed in Canada with commensurate increases [42,51,52,53,54]. These increases are not a simple result of more testing, but rather, the widespread nature of this infection [21]. This alarming trend has prompted organizations such as the Centers for Disease Control and Prevention (CDC), World Health Organization (WHO), and the Canadian Cancer Society (CCS) to label the rising incidence of oropharyngeal cancer as an epidemic. However, although the incidence of HPV-negative OPC has decreased by 50% over the last three decades, the overall incidence of OPC has increased by 28%, an increase attributed to HPV infection [21,48,54]. Further, OPC seems to disproportionately affect men. It is, however, still unknown why this discrepancy exists for HPV-positive head and neck cancers, considering relatively equal rates between genders for participation in oral sex acts. In fact, within the United States, while differences by age group do exist, it has been reported that approximately 85.4% of men and 83.2% of women have performed oral sex [55].

#### 1.2.4. Introduction of the HPV Vaccine

In 2006, Health Canada, along with the Food and Drug Administration (FDA) in the United States, approved the use of a quadrivalent HPV vaccine (Gardasil™, Merck Canada, Inc., Kirkland, QC, Canada) to protect against HPV strains 6, 11, 16, and 18. A subsequent bivalent vaccine (Cervarix™, GSK Canada, Mississauga, ON, Canada) was approved in 2009, with this vaccine protecting against HPV strains 16 and 18 only. Gardasil™ was initially recommended for use in females between 9 and 13 years old to prevent cervical lesions. Health Canada also recommended that females up to the age of 26 receive the vaccine, even if they had already been sexually active. The efficacy of the vaccine for preventing cervical lesions had previously been found to be greater than 95% effective [56]. In 2009, Gardasil™ was approved for use in males as a means to prevent genital warts, and in 2010, its use was added for prevention of anal cancer [57]. Further, in 2011, the Advisory Committee on Immunization Practices of the FDA recommended that Gardasil™ be routinely used in males aged 11–12 [43]. Currently available vaccines (i.e., Cervarix™, Gardasil™, and Gardasil9^TM^) provide a very high prevention rate for cervical and anal HPV 16 or 18 infections [56,58], with Gardasil9^TM^, which was introduced in 2014, addressing nine specific subtypes of HPV including some related to head and neck cancers.

Work by Zhang and colleagues has reported projections (2020–2045) which suggest that while HPV vaccinations may reduce OPC in younger people in future years, they will increase in those who are older [59]. It also has been estimated that this increase in OPC in men will be approximately 3% per year in the US [59,60]; however, knowledge remains poor [61,62,63].

#### 1.2.5. Reasons for Non-Vaccination

A variety of explanations for the low rates of HPV vaccination have been cited in the literature. The role of the parent(s) becomes paramount in deciding whether or not to vaccinate children. Many parents simply do not want to talk to their adolescent children about sexually transmitted infections; similarly, other parents feel that by vaccinating their children they are somehow promoting promiscuity [64], although follow-up studies have proven this fear to be groundless [65,66]. Additionally, the so-called “anti-vaccination” movement (a movement that increased dramatically in the era of the coronavirus pandemic of 2020) has become increasingly prevalent. Yet, one of the most significant reasons for non-vaccination is the lack of knowledge people have regarding HPV, HPV risk factors, its potential consequences, and the HPV vaccine itself [67,68]. Therefore, issues related to awareness and knowledge of HPV are critical.

From a population standpoint, it is known that the prevalence of HPV infection is highest among people under the age of 25. It is also known that 85% of males and 82% of females will have engaged in sexual activity by the time they are 25 years of age [69]. Therefore, having an accurate sense of what this general young adult population (age 18–24) knows about HPV, HPV-related cancers, and the HPV vaccine has important implications for educators, policy makers, and health care providers tasked with preventing HPV-related morbidity and mortality [70]. Based on comprehensive data, it appears that a reduction in the incidence of HPV will be closely tied to the acceptance of vaccination [21].

### 1.3. Awareness and Knowledge of HPV, HPV-Related Cancers, and the HPV Vaccine

The term “awareness” refers to one’s yes/no acknowledgment of ever having heard of something. The term “knowledge” refers to one’s understanding of specific facts relating to the person, place, or thing in question. Therefore, awareness is a necessary precursor for knowledge; one cannot have knowledge of HPV without first having heard of HPV. However, one *can* be aware of HPV without having any knowledge of it, as knowledge exists on a continuum ranging from no knowledge to a high level of understanding.

Many studies have evaluated the awareness and knowledge levels of HPV in various populations. HPV awareness levels can range from as low as 10% in a large sample of over 10,000 Danish men of all ages [71] to over 95% of the population in samples of female university students [72]. Similar variability has been reported for HPV vaccine awareness, ranging from 63% awareness in a sample of American males [73] to 87% awareness in the general population [74] to over 95% in populations of female university students [75].

#### Knowledge

Knowledge of HPV and the HPV vaccine is also highly variable depending on the population studied. In a systematic review that assessed HPV knowledge in over 20,000 individuals across multiple countries, correct responses to questions regarding common facts about HPV varied widely. For example, between 8–68% of respondents knew HPV was a risk factor for cervical cancer, between 10–73% of respondents knew that HPV can be asymptomatic, and between 47–87% of respondents knew HPV is sexually transmitted [76]. In general, women tend to know more about HPV than do men [76]; however, the fact remains that many women also have a very limited understanding of HPV and how it relates to cancer [77].

### 1.4. Influence of Demographic Variables

Many studies have shown significant racial and ethnic differences in HPV awareness and knowledge. In a study by Joseph et al. [78], only 42% of African-American individuals identified HPV as a risk factor for cervical cancer, compared to 90% of Caucasians. Racial disparities in awareness and knowledge of HPV and the HPV vaccine also have been reported in samples of women only [79]. Differences in knowledge between publicly and privately insured women have also been reported, with significantly higher knowledge levels observed in privately insured women, suggesting a potential link between socio-economic status (SES) and HPV knowledge [80]. Thus, SES, cultural background, level of education, and ethnicity have been associated with differences in HPV knowledge levels [76,78,81,82]. People who are younger, female, and who have more education are significantly more likely to have awareness of the HPV vaccine [83] and increases in awareness and knowledge are also strongly correlated with the intention to be vaccinated [84,85], the adoption of health-protective behaviors [86], and vaccine uptake [87,88,89,90,91].

Based on the collective data, HPV infection is an extremely important health issue, and HPV-related morbidities have become increasingly prevalent in recent decades [1,21,32,33,50,92,93]. However, there is wide variation in general awareness of HPV and in overall HPV knowledge levels (i.e., what people actually know about it). Furthermore, few studies have assessed knowledge of HPV or the HPV vaccine as it relates to non-cervical cancers, particularly in respect to head and neck cancer in general and oropharyngeal cancer specifically [25]. Due to the importance of knowledge as a precursor for the prevention of HPV infection through health-protective behaviors, including vaccination, understanding the awareness and knowledge levels of young adults most at risk for HPV infection becomes paramount. Thus, the objective of the current study assessed and quantified awareness and knowledge of HPV, HPV-related cancer, and the HPV vaccine in a population of young adult university students. In doing so, we sought to identify knowledge gaps in young adults’ understanding of HPV, OPC, and the HPV vaccine through survey methodology, as well as to identify demographic variables that may lead to greater or lesser levels of awareness and knowledge specific to the HPV.

## 2. Methods

### 2.1. Participants

In total, 1005 individuals participated in this study. Participants ranged in age from 18 years, 0 months to 30 years, 11 months (mean = 20.92 years). Of these, 711 were female, 292 were male, and two participants self-identified as being of non-binary gender. Female participants were slightly younger (mean age 20.84 years, range = 18–30) when compared to their male counterparts (mean age 21.12 years, range = 18–30). All participants were current students at a large public Canadian university located in the province of Ontario. The sample population included students from multiple faculties. Because participation was voluntary, and provided that potential participants met the inclusion/exclusion criteria, this was a sample of convenience. A summary of the number of participants from each faculty can be found in Table 1.

### 2.2. Participant Recruitment

All participants were recruited in one of three ways: first, a member of the research team approached a potential participant in common areas of the university campus and provided a brief verbal description of the study. If interested, the potential participant provided their email address. Within 24 h, a letter of information for the study, which contained a link to the online survey, was emailed to them. A reminder email was sent to all participants who had not responded within 7 days since original recruitment. Interested participants could also choose to complete the survey at the time of contact with the researcher using a tablet device connected to Wi-Fi internet. As a second recruitment option and after obtaining permission from class instructors, verbal announcements were made in various university courses at the end of lectures. These announcements consisted of a short (approximately two minutes) description of the study objectives and the task requested. Potential participants were instructed to email the researcher directly if interested. Upon receiving emails from potential participants, the letter of information for the study along with a direct link to the online survey were sent to them within 24 h. In conjunction with recruitment method 2, and with the instructor’s permission, an announcement also was posted to course websites for classes in which a verbal announcement was made. This announcement contained a reminder about the verbal announcement made in class and an attached letter of information with a link to the survey. Prior to the initiation of this research study, the Ethics Review Board approved this protocol (REB Approval #105733).

#### 2.2.1. Inclusion Criteria

Participants had to be currently registered as students. Both undergraduate and graduate students were included, and all were required to be between the ages of 18 and 30 and native English speakers. This population was chosen because they represent the age cohort with the highest prevalence of both cervical and oral cavity HPV infection [15,16,94].

#### 2.2.2. Exclusion Criteria

Individuals younger than 18 or older than 30 years of age were excluded from this study. These exclusion criteria were based on the judgement that individuals over the age of 30 represent a different cohort than that of traditional “university aged” young adults. Further, both males and females over age 30 also fall outside of the age group that is at the highest risk of acquiring an HPV infection [15,26] and were either above or near the upper limit of the recommended age for the HPV vaccine [95,96].

### 2.3. Procedure

This study consisted of a cross-sectional, self-administered, web-based survey design. A proprietary questionnaire was designed and utilized for data collection purposes with the intent of assessing awareness and knowledge of the HPV, oropharyngeal cancer (OPC), and the HPV vaccine (please see Supplementary Material). The researcher-designed questionnaire was designed and administered through the website Surveymonkey.com. In compliance with ethical requirements, informed consent was indicated by the participant’s voluntary completion of the questionnaire. The entire survey typically required from three to six minutes to complete.

#### 2.3.1. Measurement Instrument/Questionnaire

The questionnaire utilized for this study was a 42-item, proprietary survey consisting of four sections: (1) Demographic Information, (2) HPV Awareness and Knowledge, (3) OPC Awareness and Knowledge, and (4) HPV Vaccine Awareness and Knowledge. Individual survey items were adapted and modified from a total of seven prior studies. Five of these studies focused on HPV knowledge and awareness and the vaccine [74,97,98,99,100,101], while the other two identified risk factors for OPC [53,102]. Questions from the two OPC risk factor studies were transformed into a series of “true/false/I don’t know” questions in order to assess participants’ OPC knowledge. Individual items included in the questionnaire were adapted from the aforementioned literature and selected for inclusion in the present study based on their relevance to our objectives (see Addendum A). Prior to initiation of data collection, 10 individuals who did not participate in this study (5 graduate students and 5 undergraduate students) examined the questionnaire for face and content validity and provided feedback for modification to the research team.

#### 2.3.2. Demographic Information

The demographic section consisted of seven items: participant’s age, gender, ethnicity, current level of education, faculty in which they were enrolled, HPV vaccination status, and primary source of HPV information. Age was measured in years plus the closest number of additional months since one’s last birthday (e.g., 20 years, 7 months). Ethnicity categories were sourced from Canadian census recommendations, and participants were able to specify ”other” if they did not identify with any of the ethnic categories listed. Current level of education was classified by year of study (i.e., first, second, third, fourth, fifth year undergraduate or graduate student), and all graduate students were classified to be in the same category of education level, regardless of their year. HPV vaccination status was assessed by the number of vaccine doses received; participants could also select “not vaccinated” or “I don’t know” in reference to vaccination status.

#### 2.3.3. HPV Awareness

HPV awareness was assessed with a single yes/no question, “*Prior to this survey*, *have you ever heard of the Human Papillomavirus (HPV)?*”. If the participant answered ”yes” to this question, they would proceed to the HPV knowledge questions. If the participant answered ‘no’ they would go directly to the next section, which addressed OPC awareness and knowledge. This method of question administration assumed that if the participant had never previously heard of HPV, they would also know nothing about it. Therefore, to prevent these participants from providing responses to questions for which they had no knowledge, those who indicated that they had not heard of HPV before never had access to the HPV knowledge questions.

#### 2.3.4. Perceived Concern of HPV Infection

All participants rated their own personal level of concern about potentially becoming infected with HPV. This rating was made using an equal-appearing interval (EAI) scale that ranged from 1 to 5 (a response of 1 meant that the participant was “not concerned at all”, while a response of 5 indicated that the participant was “extremely concerned”).

#### 2.3.5. Self-Perceived HPV Knowledge

Participants also rated their self-perceived level of HPV knowledge on a second EAI scale. A response of 1 meant the participant believed that they knew “nothing” about HPV, with a response of 5 representing “very much/expert”, thus indicating that the participant thought their knowledge levels were considerable.

#### 2.3.6. HPV Knowledge

The HPV knowledge section consisted of 17 statements regarding established facts about HPV and HPV risk factors. After reading each statement, participants could choose the responses “true”, “false”, or “I don’t know”. The “I don’t know” option was once again included in an effort to reduce or prevent participants from guessing; participants were explicitly instructed to choose this option if they would consider their answer to be a guess.

#### 2.3.7. Oropharyngeal Cancer (OPC) Awareness

OPC awareness was assessed with a single “yes/no” question: “Prior to this survey, have you ever heard of oropharyngeal cancers (cancer of the throat, base of tongue, soft palate, and/or the tonsils?)”. If the participant answered “yes” to this question, they would proceed to the OPC knowledge questions; if they answered “no”, they would go directly to the next section (HPV vaccine awareness and knowledge).

#### 2.3.8. Self-Perceived Concern of Developing Oropharyngeal Cancer

All participants rated their level of concern about developing oropharyngeal cancer using a 5-point EAI scale. A response of 1 meant that the participant was “not concerned at all”, while a response of 5 meant that the participant was “extremely concerned” about developing OPC.

#### 2.3.9. Self-Perceived Oropharyngeal Cancer Knowledge

Similarly, all participants were asked to rate their self-perceived level of oropharyngeal cancer knowledge using an EAI scale. A response of 1 meant that the participant knew “nothing” about OPC, while a response of 5 (“very much/expert”) indicated that the participant had considerable knowledge.

#### 2.3.10. Oropharyngeal Cancer Knowledge

The OPC knowledge section consisted of six questions for which either a true/false or “I don’t know” response was required. These questions addressed OPC risk factors (e.g., “Smoking tobacco increases the risk of developing oropharyngeal cancer”), incidence trends (e.g., “The number of new oropharyngeal cancer cases per year is increasing”), and general OPC-related facts (e.g., “Both men and women can get oropharyngeal cancer”).

#### 2.3.11. HPV Vaccine Awareness

This section followed the same format as the previous two sections. HPV vaccine awareness was assessed with the yes/no question: *“Prior to this survey*, *have you ever heard of the HPV vaccine (brand names Gardasil™ or Cervarix™)?”*. An answer of “no” would take the participant to the end of the survey. If the participant answered “yes”, they would proceed to the HPV vaccine knowledge questions.

#### 2.3.12. HPV Vaccine Knowledge

The HPV vaccine knowledge section consisted of five statements to which participants could once again respond with one of three options: “true”, “false”, or “I don’t know”. Items in this section included statements regarding the vaccine’s function (e.g., “The HPV vaccine protects against cervical cancer”) and who can receive the vaccine (e.g., “Men cannot obtain the HPV vaccine”).

### 2.4. Data Analysis

Raw data from individual survey responses were exported from Surveymonkey.com into a Microsoft^®^ Excel 2021 spreadsheet. Descriptive statistics (i.e., means, medians, standard deviations, and ranges) were calculated for the demographic data. Descriptive statistics were also used to summarize the responses to the three awareness questions (e.g., total number of people who had heard of HPV, OPC, and the HPV vaccine).

A knowledge score for each of the three knowledge sections was generated using the number of correct responses within each section. Therefore, a participant’s knowledge score could range from 0–17 in the HPV knowledge section, 0–6 in the OPC knowledge section, and 0–5 in the HPV vaccine knowledge section. All responses of “I don’t know” were counted as incorrect. Participants who skipped a knowledge section due to an answer of “no” on the preceding awareness question were given a knowledge score of zero for that section. As noted previously, this followed the logic that if, for example, participants had no awareness of HPV, then they also would not have knowledge related to it. Upon completion, the number of correct responses to all 28 knowledge questions was summed to create a total knowledge score for each participant (i.e., the total knowledge score could range from 0–28).

Descriptive statistics were also used to report the percentage of participants who answered each individual question, allowing for the identification of specific knowledge gaps in this sample. Overall levels of knowledge were determined using the combined mean scores of each participant for each of the three knowledge categories. A mean total knowledge score was calculated by determining the average number of correct responses to all 28 knowledge questions from all participants. Comparisons were made between different demographic groups using these mean knowledge scores.

### 2.5. Comparison of Knowledge Scores Between Genders

Four independent t-tests were performed [103] to compare the HPV, OPC, HPV vaccine, and the total mean knowledge scores between men and women. An a priori alpha level of *p* ≤ 0.05 was used in order to determine significance. However, because four *t*-tests were performed, a correction in the alpha level of 0.05 was divided by four to further decrease the probability of a Type 1 error. Therefore, an a priori significance level of 0.0125 was used for significance.

### 2.6. Correlation Analyses of Knowledge Scores

A Pearson’s product-moment correlation coefficient was performed [103] in order to determine potential relationships between the four knowledge scores (HPV, OPC, HPV vaccine, and total score). Male and female knowledge scores were correlated separately. Demographic variables such as age and year of study were also included in the correlational assessment relative to the scores obtained.

## 3. Results

### 3.1. Response Rates

In total, 454 individuals were approached directly using Recruitment Method 1. Of these, 198 completed the survey at that time using a tablet. The remaining 236 individuals responded via email, of which 119 (50.4%) completed it. Twenty people refused participation altogether. Overall, 317 (69.8%) of the 454 people approached using this method of recruitment completed the survey.

In addition to Recruitment Method 1, 45 class announcements were made using Recruitment Method 2. Based on the number of students enrolled in each class, and assuming all students were present, it was estimated that 4985 students were exposed to this method of recruitment; it is, however, possible that some potential participants were enrolled in multiple courses in which announcements were made. In total, responses from 733 participants were collected using this method of recruitment (a response rate of 14.7%). Therefore, based on both methods of recruitment, 5439 students were exposed to at least one method of recruitment and 1050 responses were gathered with an overall response rate of 19.3%.

### 3.2. Participant Response Exclusions

In total, 45 of 1050 responses were excluded from data analysis. Nineteen were excluded due to being 30 years of age or older, three were excluded for missing responses on one section of the survey and twenty-three were excluded due to missing responses on multiple sections. Therefore, a total of 1005 responses were included in final data analysis.

### 3.3. Demographic Information

#### 3.3.1. Gender

In total, 711 females (mean age = 20.84 years, range = 18 years, 0 months–30 years, 0 months), 292 males (mean age = 21.12 years, range = 18 years, 0 months–30 years, 10 months), and 2 non-binary (mean age 20.5 years) individuals were included in final data analysis. “Non-binary” included those who were transgender, agender, bigender, gender-fluid, and others. The proportion of males (29%) to females (71%) differed slightly from the overall full-time undergraduate and graduate student body, which was reported to comprise 45% males and 55% females. Non-binary individuals were not included in gender comparisons; however, they were included in other demographic comparisons (year and program of study, etc.).

#### 3.3.2. Age

**Figure 1:**
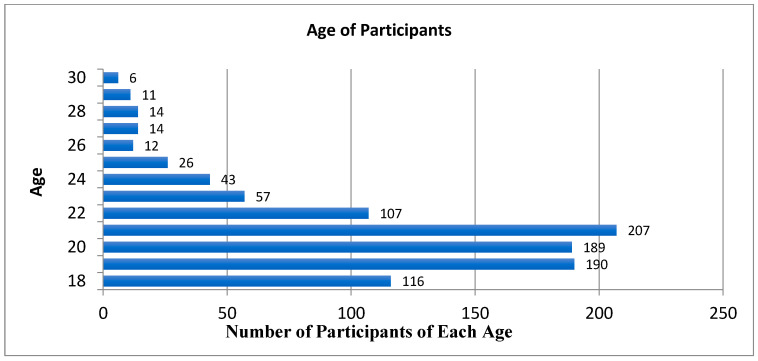
Age of participants. Note: 13 participants did not specify their age.

The mean age of all 1005 participants was 20.92 years (range = 18 years, 0 months–30 years, 10 months); 13 participants did not specify their age (Figure 1).

#### 3.3.3. HPV Vaccination Status

**Figure 2:**
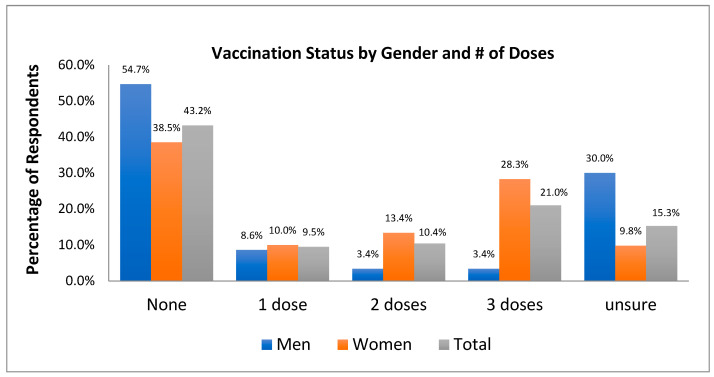
Participant vaccination status.

Data indicate that 412 (40.9%) respondents had received *at least one dose* of an HPV vaccine. Vaccination rates were much higher in females, with 51.6% of females (*n* = 367) and 15.4% (*n* = 45) of males having received at least one dose. A total of 30% of males (*n* = 85) and 9.8% of females (*n* = 67) were unsure of their vaccination status (Figure 2).

### 3.4. HPV Knowledge Sources

**Figure 3:**
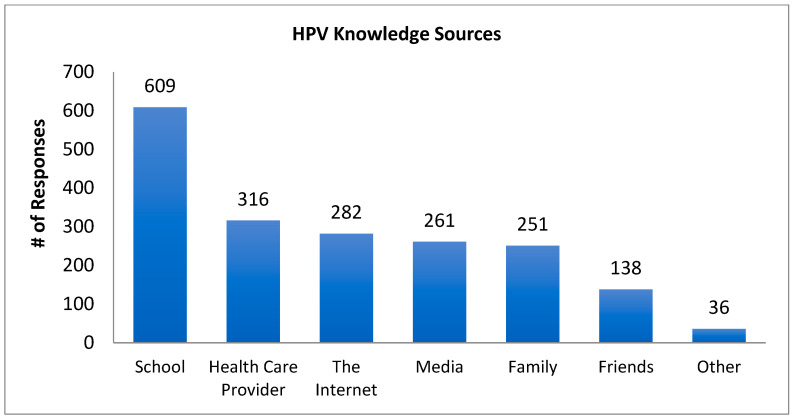
HPV knowledge sources.

Participants were asked where they had obtained information regarding HPV and they were permitted to select more than one option if applicable (Figure 3).

### 3.5. HPV Awareness

Data revealed that 92.94% (*n* = 933) of participants had heard of HPV and 5.07% (*n* = 51) had not. HPV awareness was slightly higher for females (95.07%) than for males (87.33%).

### 3.6. Perceived Concern of HPV Infection

**Figure 4:**
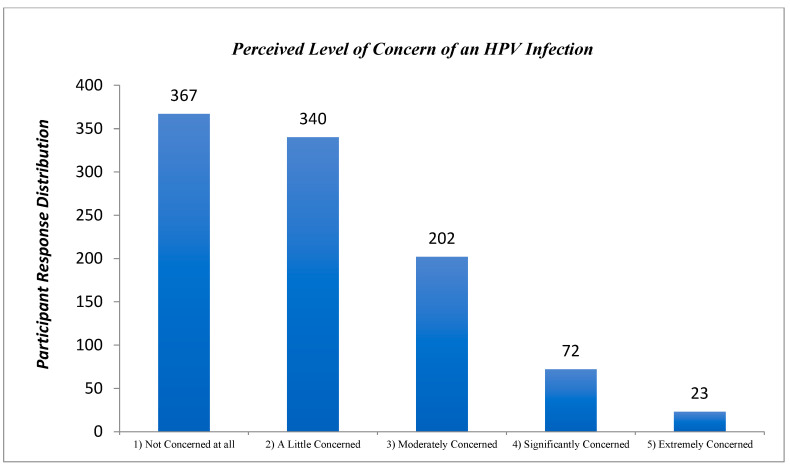
Perceived level of concern about HPV infection.

The majority of participants (90.4%) rated their level of concern as “moderately concerned” or lower, 72 (7.1%) rated their level of concern as “significantly concerned”, and 23 (2.2%) rated their concern as “extremely concerned” (Figure 4).

### 3.7. Self-Perceived HPV Knowledge

**Figure 5:**
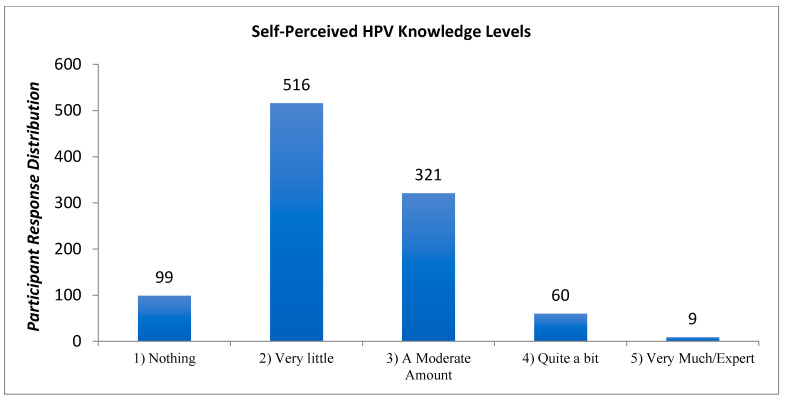
Self-perceived HPV knowledge levels.

A majority of respondents (51.3%) perceived themselves as having “very little” knowledge of HPV. A summary of participant responses to this question is shown in Figure 5.

### 3.8. HPV Knowledge

**Figure 6:**
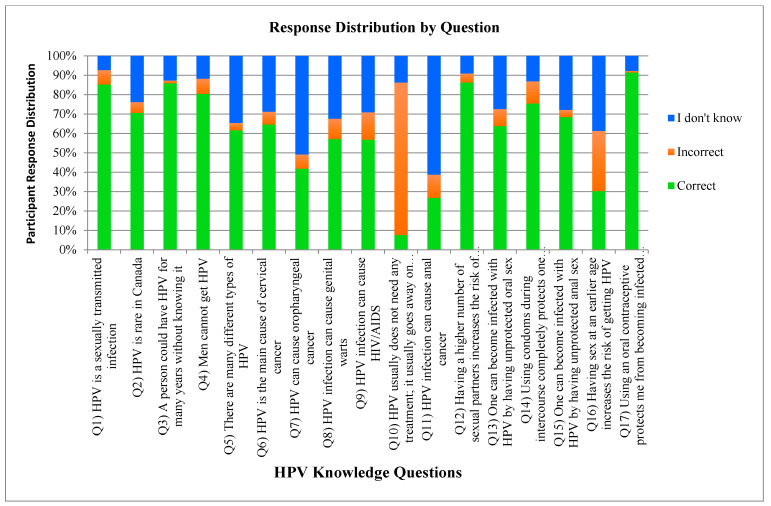
Distribution of responses to HPV knowledge questions.

In total, 954 participants (259 males, 693 females, 2 non-binary) fully completed the HPV knowledge section of the survey. However, 51 (5.3%) participants were excluded from further analysis because they indicated that they were unaware of HPV (Figure 6).

### 3.9. HPV Knowledge Scores

Of the 954 participants who completed this section, the mean HPV knowledge score was 10.54, with a maximum score = 17 (SD = 3.44, Range = 0–17, Median = 11). Knowledge scores were slightly higher for females (10.62, SD = 3.33, Range = 0–17, Median = 11) compared to males (10.39, SD = 3.68, Range = 0–17, Median = 11).

### 3.10. Oropharyngeal Cancer (OPC) Awareness

Specific to cancer awareness, 77.51% (*n* = 779) of participants reported having heard of OPC prior to completing the survey, yet 21.29% (*n* = 214) had no prior knowledge, and 1.19% (*n* = 12) did not respond to this question. OPC awareness was approximately 7% higher for females than males, with 78.89% of females and 71.92% of males indicating awareness of OPC.

### 3.11. Self-Perceived Concern of Developing Oropharyngeal Cancer

**Figure 7:**
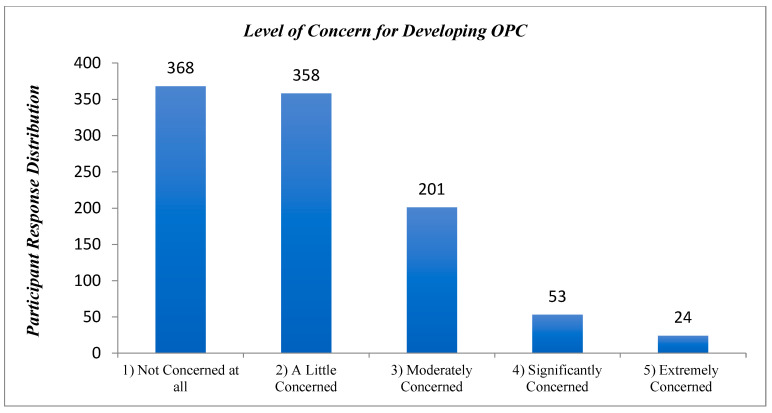
Perceived level of concern about developing OPC.

Most participants indicated that they were “not concerned at all” (*n* = 368) or “a little concerned” (*n* = 358) about contracting OPC (Figure 7).

### 3.12. Self-Perceived OPC Knowledge

**Figure 8:**
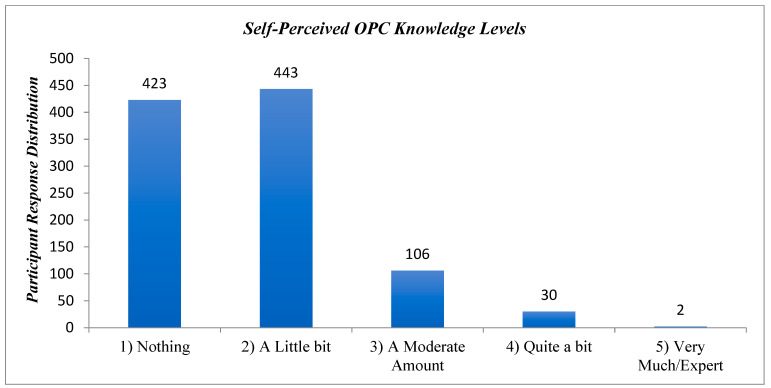
Self-perceived OPC knowledge levels.

The participants’ self-perceived level of OPC knowledge revealed that the vast majority either knew “nothing” (*n* = 423) or only “a little bit” (*n* = 443), while 138 participants (13.7%) indicated they had a moderate or greater amount of knowledge (Figure 8).

### 3.13. OPC Knowledge

**Figure 9:**
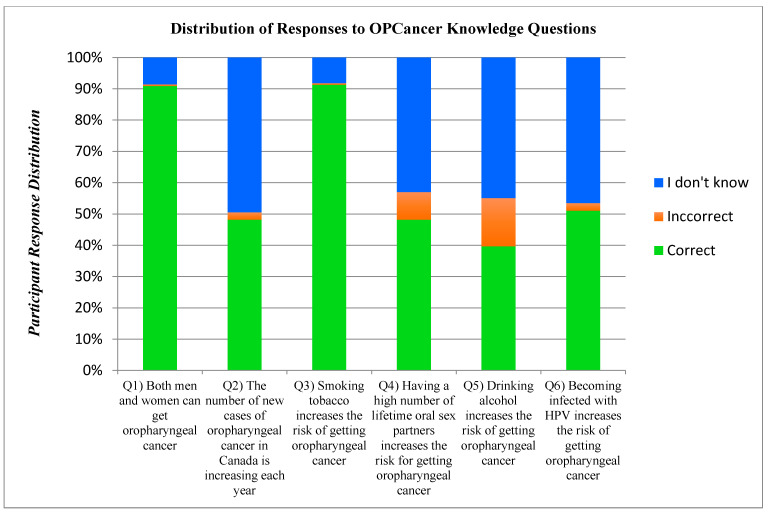
OPC knowledge questions and responses.

In total, 791 participants (576 females, 214 males, 1 non-binary) completed this section. Interestingly, 214 (21.29%) participants were excluded from this section because they had not heard of OPC (Figure 9).

#### OPC Knowledge Scores

Of 791 participants who completed this section, the mean OPC knowledge score was 3.69 (SD = 1.49, Range = 0–6, Median = 4). Again, knowledge scores were slightly higher for females (3.70, SD = 1.45, Range = 0–6, Median = 4) when compared to males (3.67, SD = 1.57, Range = 0–6 Median = 4).

### 3.14. HPV Vaccine Awareness

A total of 87.86% (*n* = 883) of participants indicated that they had heard of the HPV vaccine, and vaccine awareness was 17.28% greater for females (92.97%) than for males (75.69%). However, 119 (11.84%) participants (49 females, 69 males, 1 non-binary) reported that they had no knowledge of the HPV vaccine.

### 3.15. HPV Vaccine Knowledge

**Figure 10:**
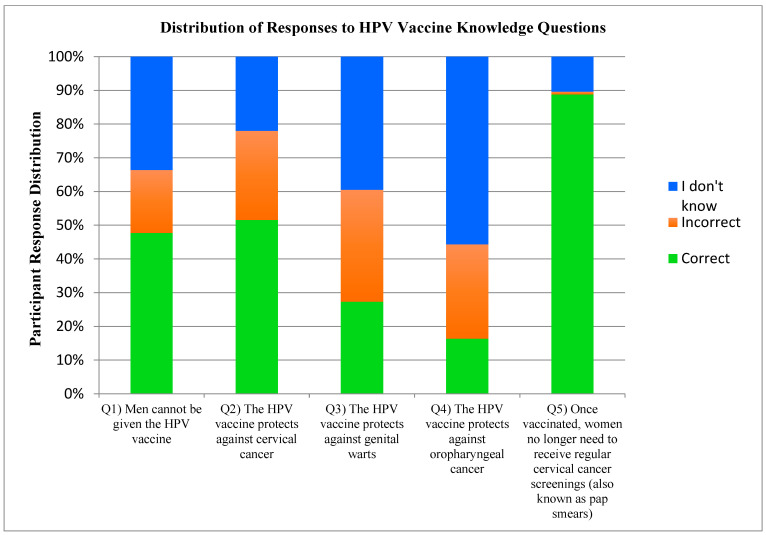
HPV vaccine knowledge questions and responses.

In total, 886 participants completed the HPV vaccine knowledge section (662 females, 223 males, 1 non-binary); 119 participants were excluded because they had never heard of the vaccine. A summary of the response distribution for each HPV vaccine knowledge question is presented in Figure 10.

#### HPV Vaccine Knowledge Scores

Of 886 participants who completed this section, the mean vaccine knowledge score was 2.32 out of 5 (SD = 1.23, Range = 0–5, Median = 2); knowledge scores were slightly higher for females (2.33, SD = 1.18, Range = 0–5, Median = 2) when compared to males (2.29, SD = 1.38, Range = 0–5, Median = 2).

### 3.16. Total Knowledge Scores

The mean total knowledge score (maximum score = 28) was 14.95 (SD = 6.12, Range = 0–28, Median = 15). Females had greater total knowledge scores (15.51, SD = 5.59, Range = 0–28, Median = 16) compared to males (13.66, SD = 7.04, Median = 14). Given that participants could access each section’s knowledge questions only if they answered “yes” to the preceding awareness question, not all participants had access to all 28 questions. Thus, those who answered ”no” to one or more awareness questions could not achieve a full knowledge score.

### 3.17. Demographic Factors, Awareness, and Knowledge

*Year of Academic Study.* Seven participants did not specify their year of education and were therefore excluded. Awareness levels and mean knowledge scores were calculated for each cohort and for each section of the questionnaire (HPV, OPC, and HPV vaccine). Awareness levels were calculated by determining the percentage of participants in each cohort who had previously heard of HPV, OPC, and the HPV vaccine, respectively. Mean knowledge scores for each section were generated using only the responses of participants who had either answered ”yes” to the corresponding awareness question or left the awareness question blank. Thus, participants who answered ”no” to the question “*Have you ever heard of HPV prior to this survey?*” were not included in the calculation of mean HPV knowledge scores. This process also applied for the OPC and HPV vaccine sections (Table 2).

### 3.18. Program of Study

Awareness levels were calculated by determining the percentage of participants in each cohort who had previously heard of HPV, OPC, and the HPV vaccine, respectively. Mean knowledge scores for each section were generated using only the responses of participants who had either answered ”yes” to the corresponding awareness question or left the awareness question blank, thereby granting them access to the knowledge questions (Table 3).

### 3.19. Ethnicity

Due to a high number of Caucasian participants (73.6%) and the relatively small number of respondents in other ethnicity cohorts, participants were grouped into one of two categories for comparison: Caucasian (White) or Other. Awareness levels and mean knowledge scores were generated in the same manner as the previous demographic variables (Table 4).

### 3.20. Vaccination Status, Awareness and Knowledge

Participants were categorized into three categories based on their HPV vaccination status: (1) not vaccinated, (2) vaccinated, or (3) unsure. Participants were deemed vaccinated if they had received at least one dose of the HPV vaccine (either Gardasil™ or Cervarix™). Five participants did not indicate their vaccination status and were excluded. Awareness levels and mean knowledge scores for each section of the questionnaire are presented in Table 5.

### 3.21. Comparison of Mean Knowledge Scores Between Genders

Gender was not found to be statistically significant in relation to either HPV knowledge (t = 0.856, *p* = 0.393), OPC knowledge (t = 0.214, *p* = 0.830), or HPV vaccine knowledge (t = 0.352, *p* = 0.725). However, the total knowledge score was found to be statistically significant between females and males (*p* = 0.0125, t = 4.009, *p* = 0.000).

### 3.22. Correlational Analyses of Knowledge Scores

Results for both males and females indicated strong correlations between HPV knowledge scores and total knowledge scores. Strong correlations also were noted between OPC knowledge and total knowledge and HPV vaccine knowledge and total knowledge. Weak-to-moderate correlations were found between the three knowledge section scores (HPV to OPC, OPC to HPV vaccine, and HPV to HPV vaccine). However, for males, a moderate correlation was noted between HPV knowledge and HPV vaccine knowledge. Therefore, males who had higher HPV knowledge scores also had higher HPV vaccine knowledge scores. No correlation was found between age or year of academic study and knowledge scores. The correlation matrices for females and males are shown in Table 6 and Table 7, respectively.

## 4. Discussion

This study assessed awareness and knowledge of HPV, HPV-related cancer, and the HPV vaccine in a population of young adult university students (18–30 years of age). This included identifying knowledge gaps in their understanding of HPV, OPC, and the vaccine through a series of “true/false/I don’t know” questions and identifying demographic variables that may lead to greater or lesser levels of awareness and knowledge specific to the HPV-related topics. Details of these findings will be discussed in subsequent sections.

### 4.1. Response Rates and Participant Demographics

Specific to recruitment, approaching individuals directly yielded a much higher response rate (69.8%) compared to in-class recruitment (14.7%). Overall, however, the present investigation yielded a response rate that is consistent with previous research [104].

*Gender.* Seventy-one percent of the current respondents were female and twenty-nine percent were male. Given the significant difference in the total knowledge scores between males and females, it is plausible that overall knowledge levels may have decreased slightly had there been a higher proportion of males. This assumption is based on the fact that males had a significantly lower total knowledge score than females.

*Ethnicity.* While no method of categorizing ethnicity is fully accurate, the census categories were chosen because they represent the best current approach for categorizing one’s ethnic identity. Accordingly, a majority of participants (74%) self-identified as Caucasian.

*Faculty of Participant’s Enrollment.* Health Sciences accounted for the largest proportion of respondents (36.5%), followed by Social Science (13.5%) and Science (12%). Although faculties were not evenly represented in this sample, there is no reason to assume that students from one faculty would have inherently better or worse HPV, OPC, HPV vaccine knowledge, and/or awareness. Our assumption is supported by the data, which show only minor differences in awareness and knowledge levels between respondents from different faculties.

*HPV Vaccination Status.* Almost 41% of respondents indicated that they had received at least one dose of the HPV vaccine. This overall vaccination rate comprised 51.6% of female respondents and 15.4% of male respondents. The vaccination rate for females in the present sample is lower when compared to the reported vaccination rate of 62–67% coverage with at least one dose in 2021. Thus, the females who participated would have been exposed to the HPV vaccination program at a time when the vaccine was newer and when coverage rates were lower (e.g., 50% coverage in 2007), as per Wilson et al. [105]. This finding suggests that as vaccination rates continue to rise in females, and as these females subsequently become older, vaccination coverage rates will naturally increase. The likely effect of this trend would then ideally manifest as a decreased HPV infection prevalence. Obviously, efforts that seek to monitor such future trends would be valuable relative to understanding associated increases in HPV awareness and knowledge.

*Year of University Study.* The distribution of participants based on year of study was relatively even, although a smaller proportion of students in their first year of study was represented. As some students in their first year of study are potentially under the age of 18, some may have been excluded from participating due to age. However, it is unclear how many potential participants this may have affected.

In general, knowledge and awareness seemed to improve with one’s year of study; the lowest awareness levels and lowest mean knowledge scores were found in those who were in either their first or second year of study. This finding was true across all three knowledge categories (HPV, OPC, and HPV vaccine), suggesting that level of education may play some role in one’s increasing knowledge and awareness of HPV, OPC, and the HPV vaccine. This finding may support past work which indicates that educational level is significantly associated with awareness [82].

*HPV Awareness.* A high rate of participants (92.94%) had heard of HPV prior to participation, and this level of awareness is consistent with previous research. Although primarily focusing on females, prior studies have reported awareness rates of between 94% and 96% [72,106]. Our findings of higher awareness levels in females (95.07%) vs. males (87.33%) is also consistent with the past findings [82,107,108]. Again, exposure to information on HPV and the vaccine, as well as associated advertising and educational materials for females, is likely an influencing factor. If males had more exposure to information or greater opportunities to receive the vaccine, their HPV awareness might begin to approximate that observed in our females.

Although those who identified as Caucasian had higher levels of HPV awareness (94.73%) than did non-Caucasians (87.55%), the representation of these two groups differed. This finding is consistent with previous research which has linked Caucasian ethnicity as a significant predictor of higher HPV awareness levels [108]. While more aware of HPV, a national sample of adults in the United States revealed that Caucasian individuals also engage in oral sex practices more often than those of other races [69,109]. Interestingly, that work also noted that having a higher level of education was also related to having two or more vaginal sexual partners within the prior year for males and more frequent oral sex for both men and women [69].

*HPV Knowledge.* Although a relatively high level of general awareness was present in our sample, HPV knowledge varied greatly by knowledge item. As noted, HPV knowledge was assessed through 17 “true/false/I don’t know” questions. In total, 954 participants completed the HPV knowledge section, and the mean summed knowledge score of these participants was 10.54 (range = 0–17), reflecting a moderate level of HPV knowledge. General facts about HPV were fairly well known, suggesting that young adults in the current sample have been exposed to at least some form of HPV education, whether or not that exposure has been provided in a formal manner. This suggestion is further supported by the fact that 60.5% of respondents reported that they had received some information or knowledge of HPV as part of a school curriculum. This exposure may explain the fact of why respondents were able to correctly answer certain HPV knowledge questions. For example, 85.2% of respondents to the HPV knowledge section correctly identified HPV as a sexually transmitted infection, while 91.4% knew that using an oral contraceptive does not protect against infection, and, finally, 85.8% knew that HPV infections could be asymptomatic for many years. Further, 80.4% of all respondents knew that men could become infected with HPV.

Similar findings have been reported by Krawczyk et al. [106] relative to university students’ knowledge that HPV is a sexually transmitted infection. The present findings, in addition to those of Krawczyk et al. [106], may represent examples of positive results of past sexual education programs. Clearly, knowledge is an important first step in the prevention of HPV as well as other types of sexually transmitted infections. It is, therefore, encouraging to find that a high proportion of those participating in our study were aware of the sexually transmitted nature of HPV. Given that sexual experimentation is a common endeavor for many university students, building on this rudimentary knowledge of HPV will be an important goal for future educators.

Certain risk factors for HPV were also fairly well known by our respondents. A majority of respondents correctly identified that having a high number of lifetime sexual partners is a risk factor for HPV, and 75% correctly noted that condoms do not completely protect against HPV infection during sexual activity. Less well known was the fact that HPV can be spread through oral and anal sex; approximately two-thirds of respondents correctly identified these behaviors as substantial risk factors. The fact that roughly one-third of respondents *did not* know that oral or anal sex may lead HPV infection should be of concern. This conclusion should be viewed in the context of a prior work which has reported that approximately 89% of women and 90% of men aged 15–44 have performed oral sex and that 36% of women and 44% of men have reported engaging in anal sex with an opposite-sex partner [55,69]. This trend has remained relatively consistent based on more current US data provided by Habel et al. [110] for the years 2011–2015.

The collective results outlined above indicate that those we sampled may possess at least a basic understanding of HPV and the associated risk factors for infection; in contrast, however, the potential consequences of HPV infection were not as well known. Slightly more than half of respondents in the present study knew that HPV can cause genital warts. These findings compare favorably to those reported by Ratanasiripong et al. [104], who assessed HPV knowledge in a sample of female college students. In that study, 43.5–46.9% of their participants knew HPV could cause genital warts. These data suggest that knowledge levels for the present sample are slightly higher relative to these two knowledge items, but overall, young adults continue to require more education relative to HPV infection [8].

Perhaps the largest knowledge gaps identified from the HPV knowledge portion of the current study were those related to the association of HPV to certain cancers. For example, only two-thirds (64.6%) of respondents who completed the knowledge section of the present survey correctly identified HPV infection as the main cause of cervical cancer. Comparatively, 41.8% of respondents identified HPV infection as a risk factor for OPC, and only 26.7% identified HPV infection as a risk factor for anal cancer. One possible explanation for these findings may be the relatively low incidence of these two types of cancer in North America. Anecdotally, it is true that more prevalent cancers such as breast, lung, colon, and prostate dominate North American culture and public interest. Interestingly, however, many of the respondents anecdotally indicated that they first learned of the link between HPV and OPC from a public figure—actor Michael Douglas—who was diagnosed with HPV-positive OPC in 2010 (https://www.theguardian.com/film/2013/jun/02/michael-douglas-oral-sex-cancer; https://oralcancerfoundation.org/people/arts-entertainment/michael-douglas/, accessed on 22 November 2024).

Based on the data obtained, our participants were largely unaware of the association between HPV and cancer, especially its relationship to OPC and anal cancer. Knowledge of HPV’s relation to cervical cancer was again similar to findings from previous samples of Canadian university students in which 61% of respondents were aware of the association [106]. Yet, other studies have shown much higher levels of knowledge in relation to the link between HPV and cervical cancer; Ratanasiripong et al. (2013) found that 86.3% of female Californian college students were aware of the link. Although we found that a higher proportion of females (69.4%) than males (52.1%) knew about the relationship between HPV and cervical cancer, females in the Ratanasiripong et al. study still demonstrated higher knowledge levels relative to ours [104].

In contrast to the above comparisons, and despite a 225% increase in the incidence of HPV-positive OPC between 1988 to 2004 in the United States, fewer studies have addressed whether people understand the association between HPV and OPC [21,48]. More than a decade ago, a national sample of men in the United States revealed that only one-in-five men were aware of this association [73]. While such awareness has increased, it remains low [111]. In fact, a recent study of US participants has revealed woefully poor knowledge of this risk [93].

The present sample revealed nearly double the level of knowledge relative to this fact, a finding comparable to a study from Italy in which 31–47% of lesbian, gay, or bisexual participants were aware of this association [99]. However, fewer than 27% of respondents of the present study knew that HPV was a risk factor for anal cancer. This was by far the least understood association between HPV and cancer. This poor knowledge of HPV as a risk factor for anal cancer is consistent with past research which reported that only 14% of men were aware of this association [73]. Although homosexual men are at an increased risk for anal cancer [112], this also should be a concern for heterosexual men and their female partners given that approximately 44% of men in the United States report having had anal sex with a female partner [48,110].

In summary, our findings confirm that while most participants had heard of HPV, significant gaps in HPV-related knowledge exist. While awareness is a necessary and crucial component of one’s understanding of HPV, it is incomplete. That is, having a more complete understanding of HPV relative to risks and potential consequences is much more likely to influence behavior than simply knowing that the virus exists. Ongoing educational efforts must address the knowledge gaps identified in this population—most notably, knowledge of the associations between HPV and cancers of the cervix, oropharynx, and anus. Efforts to educate the population about these HPV-related cancers will be imperative as the anticipated incidence of both oropharyngeal and anal cancers continue to rise [21,33].

*Oropharyngeal Cancer Awareness.* HPV-positive OPC incidence has increased substantially in developed countries such as Canada and the United States in recent decades [21,52]. This trend has prompted organizations such as the United States Centers for Disease Control and Prevention and the World Health Organization to label this trend as an epidemic. As incidence rates of HPV-related OPC continue to increase, improving awareness levels and seeking to reduce knowledge gaps via education may be highly valuable. Therefore, the current study explored awareness and knowledge relative to OPC and its risk factors. Our results revealed that 77.5% of participants had heard of OPC prior to participation. As a single independent measure, this awareness rate may be considered as good, but relatively high awareness levels may not directly translate to high levels of knowledge.

*Oropharyngeal Cancer Knowledge.* Based on our series of six “true/false/I don’t know” questions, 791 of 1005 participants completed the OPC knowledge section of the survey, and over 90% correctly identified smoking tobacco as a risk factor. However, less than half of our participants correctly identified alcohol use as a risk factor for OPC. Similarly, less than half of our respondents knew that the incidence of OPC is currently rising in developed countries [61,62,63]. With respect to OPC and its relation to HPV, only half knew that HPV infection was a risk factor and/or knew that having a higher number of lifetime oral sexual partners increased one’s risk of acquiring OPC [21]. Notably, there were no differences between males and females with respect to these two questions. Past studies that have assessed knowledge of OPC have typically focused on populations of dental professionals or other health care providers, and therefore, no direct data are available for comparison [113,114].

The relatively low level of knowledge relative to OPC and HPV speaks to a general lack of understanding about this type of cancer [61]. In fact, an overwhelming majority (86.3%) of participants in our study rated their self-perceived level of knowledge regarding OPC as either “nothing” or “a little bit”. This finding suggests that young adults may be engaging in high-risk sexual behaviors without fully understanding the direct risk related to particular sexual activities. More specifically, men are five times more likely than women to be diagnosed with an HPV+ head and neck cancer. In the context of oral sex, the viral titres found in the genital tracts of women are more than 10X higher than those found in men [109]. Thus, further efforts to educate individuals about the simple risk factors related to the relationship between oral sex practices and OPC is warranted. It has been suggested that while a reduction of HPV-related OPC does not have a clear “reversible modifiable factor to reduce risk” (p. 227), the acceptance of the HPV vaccination may reduce incidence over the next two decades [109].

*HPV Vaccine Awareness.* Our data revealed that 87.86% of participants had previously heard of the HPV vaccine (either Gardasil™ or Cervarix™). HPV vaccine awareness was much higher in females (93%) compared to males (75.7%), a result that was expected due to the fact the vaccine has been primarily marketed towards females. Our HPV vaccine awareness levels for females were consistent with findings from similar samples of female university students in both Canada and the United States who had vaccine awareness rates of 91% and 95%, respectively [104,106]. Although lower, male vaccine awareness in the present study was slightly higher than those of 63% found in a national sample of American men [73].

Because the HPV vaccine was approved for use in males more than a decade ago by the Centers for Disease Control and Prevention [43] further educational efforts should focus on increasing both awareness and knowledge of the vaccine in this group. The possibility of increasing vaccination rates in males presents a significant opportunity to increase vaccination coverage among the entire population and, subsequently, may serve to reduce the prevalence of HPV infection and its resulting morbidities. This may be especially important for men who have sex with men, as these individuals are at an even higher risk for HPV infection. However, regardless of gender or sexual practices, increasing both awareness and knowledge levels specific to HPV and its vaccine should be a high priority for educators and health care providers moving forward [70,115].

*HPV Vaccine Knowledge.* The relatively high levels of vaccine awareness did not translate to high levels of vaccine knowledge. In fact, vaccine knowledge was quite poor relative to the other knowledge sections of our survey (e.g., less than half of respondents knew that men could receive the HPV vaccine). However, prior studies have found much greater levels of knowledge; a study of Canadian adult females found that 71% of respondents knew that men could receive the vaccine [116]. Again, increasing the knowledge of this fact and the access to vaccination, especially in males, may provide an opportunity for increased vaccine uptake [91].

Perhaps the most surprising finding from our research was that only half of respondents (51.5%) knew that the HPV vaccine protects against cervical cancer, and only 27.3% knew that it protects against genital warts. These findings are surprising for two reasons. First, both vaccines currently in use (Gardasil™ and Cervarix™) were explicitly developed in order to prevent cervical cancer in females (Gardasil™ also protects against genital warts). Second, 40.9% of our participants reported having received at least one dose of the HPV vaccine, suggesting that at least some participants received the vaccine without knowing why it was administered. These findings suggest a clear distinction between awareness and knowledge about the HPV vaccine. While 87.7% of respondents had previously heard of the vaccine, only half knew the vaccine’s primary purpose. This lack of knowledge may be related to a generally poor understanding of the consequences of HPV infection. In other words, there may be confusion about the purpose of the HPV vaccine for individuals who do not know the association between HPV and cervical cancer/genital warts. Yet, it is also conceivable that the consequences are known, but that they are disregarded in the context of the commonality of sexual practices in the age group we assessed.

Finally, one knowledge question was answered correctly by a large proportion of respondents. More specifically, 88.8% of participants correctly indicated that once vaccinated, women must still receive regular cervical cancer screening; these data are consistent with those of a past study [44,45,74].

### 4.2. Influence of Demographic Variables

*Age*, *Year of University Study*, *Program of Study, and Ethnicity.* Based on the correlations generated, no relationships between age and awareness or knowledge were identified. In contrast, a clear pattern was noted between year of university study and both awareness and knowledge levels of HPV, OPC, and the HPV vaccine. However, correlational analyses revealed weak correlations between year of study and knowledge scores, but comparisons of awareness and knowledge levels between respondents in different programs of study were somewhat difficult to perform given the unequal representation of each faculty in the sample. Yet, no noteworthy differences were noted in awareness or knowledge of HPV between the programs of study. With respect to HPV vaccine awareness, respondents from Health Sciences and the Information and Media Studies had the highest levels (94% and 93%, respectively). This may be due to the fact that the majority of respondents from these faculties were female (84% and 90%, respectively). Interestingly, those enrolled in medicine and dentistry demonstrated noticeably higher levels of HPV vaccine knowledge, suggesting that these individuals likely have had greater potential exposure to this information within their prior or current education. Finally, despite variability in sample numbers across ethnicity, Caucasian individuals demonstrated higher levels of both awareness and knowledge for all categories, which is consistent with past research [78,116].

*Vaccination Status and Gender.* Surprisingly, there were minimal differences between awareness and knowledge related to HPV and the HPV vaccine between people who had been vaccinated and those who were not. However, individuals who were unsure of their vaccination status had much lower levels of awareness and knowledge. In relation to gender, females had higher levels of both awareness and knowledge for all three categories explored. While no significant differences in any of the knowledge sub-scores were observed, a significant difference between male and female total knowledge scores was identified. These findings were not unexpected given results of previous studies showing differences in knowledge levels of HPV between males and females [83,98,107,117].

### 4.3. HPV Awareness and Knowledge as an Important Factor in Prevention

Despite the extremely high prevalence of HPV infection, according to existing data, opportunities for infection prevention and subsequent prevention of the many associated morbidities still exist. The HPV vaccine will certainly play a large role in the prevention strategies of many countries; however, broad-focused sexual education and information on behavioral factors are equally important factors in combatting the high prevalence of HPV infection. The decision to engage in health-protective behaviors is also borne of having an accurate understanding of the potential consequences involved with engaging in high-risk sexual behavior. In the context of HPV infection, behavioral factors such as the decision to initiate new sexual relationships, use of protection during sex, and the type of sexual activities in which one participates almost certainly is moderated by one’s awareness, knowledge, and perceived risk of the virus.

Many health behavior theories confirm the importance of awareness and knowledge (sometimes referred to as information) as a necessary precursor to health-protective behavior [117,118,119]. These same principles apply in the decision-making process of those considering HPV vaccination for themselves or their children, as previous studies have shown a relationship between HPV knowledge levels and vaccine uptake [77,80,89,90,91,120]. Public efforts to increase awareness of the potential risks of HPV infection continue to be important, most particularly in the context of vaccination. This would most critically involve increased awareness that the age of sexual debut, as well as the increasing risks of having multiple sex partners, carries substantially greater risks [11]. Thus, the use of public information announcements, access to materials through university health care centers, and the potential capacity to educate young people earlier (e.g., during high school) may, at the very least, foster increased awareness of the inherent risks of HPV infection.

### 4.4. Implications of the Present Findings

Although a majority of HPV infections are believed to clear spontaneously with no residual effects, the number of HPV-related infections and subsequent consequences have become increasingly prevalent. Thus, having an accurate estimate of what the young adult population knows about HPV, HPV-related cancers, and the HPV vaccine has important implications for educators, policy makers, and health care providers tasked with preventing HPV-related morbidity and mortality. The present findings have clearly identified both strengths and weaknesses in young adults’ understanding of HPV, HPV-related cancers, and the HPV vaccine. Clear knowledge gaps exist in both general awareness related to HPV and more comprehensive knowledge related to its potential health consequences. Based on the present data, efforts that seek to enable future education programs to build on existing knowledge, while simultaneously seeking to address common misconceptions, are warranted.

Since knowledge is a necessary precursor to one’s adoption of health-protective behaviors, including the decision to become vaccinated, addressing knowledge gaps may allow young adults to make better choices (e.g., increasing knowledge of HPV-related morbidities may lead to safer sexual practices). Similarly, increasing vaccine knowledge, especially in males, could lead to higher vaccine coverage rates and decreased HPV prevalence, which in turn may have direct and observable health benefits.

Over 60% of our participants identified “school” as a source of their HPV information. This finding suggests that the best place to address some of the knowledge gaps identified herein is within the educational school setting. We acknowledge that controversy exists relative to whether sexual education should be part of a school curriculum, and opposition to such efforts may be substantial (e.g., discussions of sexually transmitted infections and safe sex practices). However, health care providers also may continue to play an important role in education regarding HPV and the HPV vaccine. In this regard, 31.4% of participants in this study reported a “health care provider” as a personal source of HPV information, a finding which supports the notion that a health care provider’s recommendation may be an important factor in many people’s decision to receive the HPV vaccine [70,121,122].

### 4.5. Limitations of the Present Study

The limitations of the present study must be acknowledged and considered. First, the sample obtained in this study contained a disproportionately high proportion of females. This was likely a result of a sampling or response bias that favored students enrolled in faculties where a greater number of females are enrolled. However, given the minimal differences in awareness and knowledge levels observed between individuals from different faculties, the over-representation of females may not change the findings in any meaningful way. Additionally, while Health Sciences students may be considered more “health-conscious” in general, there is no reason to assume they have a better or poorer understanding of HPV, OPC, or the HPV vaccine—or that their experiences with these subjects are any different when compared to other students within this age cohort.

Given that a significant difference in total knowledge scores was found between males and females, the high proportion of female students in this sample relative to the population may have contributed to some sampling bias with some skewing of the data. If true, it would likely result in an overestimation of actual awareness and knowledge levels, given that females were found to have higher levels of both compared to males. However, our findings are similar to previous studies that have assessed knowledge and awareness in samples of university students, and the large sample size (*n* = 1005) may increase confidence that the data obtained are representative [101].

An additional limitation of this study is the survey tool utilized. This measurement tool was designed specifically for this study, and the items were adapted from tools used in previous studies, some of which were validated. While the tool that was developed did undergo a face and content validity assessment by 10 current university students, the measure itself was not fully validated. Therefore, issues of reliability and validity need to be considered when interpreting our results.

Finally, the population studied in this research is not representative of the entire population at large. The population of university students who participated, those aged 18–30 years, was chosen due to the high prevalence of HPV infection and potentially risky sexual behavior in young adults. While the sample obtained is believed to be representative of the population in question based on the demographics of these university students, the findings of this research cannot be generalized to the population at large. This concern relates most specifically to those who are not Caucasian females.

Thus, despite the considerably large sample obtained herein, the external validity of these data must be considered carefully. Given the increased levels of awareness and knowledge found with increased education level in this study, in addition to previous studies that have reported much lower levels of awareness and knowledge in the population at large [68,71,109], awareness and knowledge levels found at present are likely higher than those of the general population. Nevertheless, this study does provide valuable insights into the current levels of awareness and knowledge relative to HPV, OPC, and the HPV vaccine in a large sample of university students. Students in this sample demonstrated good awareness of the topics in question; further, some basic knowledge of HPV, OPC, and the HPV vaccine was revealed. However, this study has clearly identified specific knowledge gaps and opportunities for future education.

Finally, strategies to address expanded educational programs and knowledge gaps identified by this research will need to be developed. Questions such as when to provide education, to whom, and in what setting will need to be addressed. Most importantly, opportunities to translate this education into behavioral action will need to be implemented [123,124]. However, given the contemporary and often misguided resistance to vaccinations in general in recent years, the ability to understand the value of vaccines in reducing HPV risk may prove to be a substantial challenge in the years ahead. Nevertheless, this issue of “how” educational opportunities can be translated into behavioral actions to reduce risk is also an essential need relative to HPV and its consequences.

## 5. Conclusions

This research investigated young adults’ awareness and knowledge of HPV, OPC, and the HPV vaccine. Based on the data gathered, several conclusions are offered. First, clear and significant gaps in knowledge of HPV, OPC, and the HPV vaccine exist in this population. More explicitly, this research showed poor understanding of the potential consequences of HPV infection. Most notably, the young adults in this sample were largely unaware of the association between HPV and cancers of the anus and oropharynx. This knowledge gap will need to be addressed, especially if the incidence of these two types of cancers continues to increase. Second, males seem to have less awareness and knowledge relative to HPV. Thus, ongoing efforts to increase awareness and knowledge in males is encouraged. Finally, this study did find that most individuals possessed some awareness and at least a basic understanding of HPV. However, educational efforts that increase awareness and knowledge of HPV, HPV-related morbidities (e.g., OPC), and a wider adoption of the HPV vaccine may ultimately lead to direct benefits in the population at large.

## Figures and Tables

**Table 1:** Demographic data of participants.

Variable	Women *n* (%)	Men *n* (%)	Total
Number of Participants	711 (71)	292 (29)	1005 *
Age (years)	Mean 20.84	Mean 21.12	Mean 20.92
Ethnicity	*n* (% of total)	*n* (%)	*n* (%)
Caucasian	522 (71)	218 (29)	740 (74)
Chinese	55 (70)	23 (30)	79 (8)
South Asian	44 (73)	15 (27)	60 (6)
Black	17 (74)	6 (26)	23 (2)
Filipino	6 (100)	0 (0)	6 (0.6)
Aboriginal	1 (100)	0 (0)	1 (0.01)
Latin American	10 (77)	3 (23)	13 (1)
Southeast Asian	10 (83)	2 (17)	12 (1)
Arab	8 (44)	10 (56)	18 (2)
West Asian	1 (100)	0 (0)	1 (0.01)
Korean	8 (80)	2 (20)	10 (1)
Japanese	2 (100)	0 (0)	2 (0.02)
Other	27 (66)	12 (34)	40 (4)
Year of Study	*n* (%)	*n* (%)	*n* (%)
1st year	94 (69)	43 (31)	137 (14)
2nd year	173 (75)	58 (25)	232 (23)
3rd year	134 (71)	56 (29)	190 (19)
4th year	152 (68)	72 (32)	224 (22)
5th year	25 (52)	23 (48)	48 (5)
Graduate	129 (77)	38 (23)	167 (17)
Faculty of Enrollment	*n* (%)	*n* (%)	*n* (%)
Arts and Humanities	35 (78)	9 (22)	45 (4.5)
Business	39 (48)	43 (52)	82 (8)
Education	0 (0)	1 (100)	1 (0.001)
Engineering	25 (30)	59 (70)	84 (8)
Health Sciences	307 (84)	57 (16)	364 (36.5)
Media Studies	54 (90)	6 (10)	60 (6)
Law	27 (73)	10 (27)	37 (4)
Music	39 (71)	16 (29)	55 (5)
Medicine and Dentistry	6 (33)	11 (66)	18 (2)
Science	81 (660)	42 (33)	123 (12)
Social Science	98 (73)	37 (27)	135 (13.5)

* Two participants self-identified as non-binary gender, seven participants did not indicate their year of study, and one participant did not indicate their faculty of enrollment.

**Table 2:** Awareness levels and mean (M), standard deviation (SD), and sample size (n) for knowledge scores by year of education.

Year of Study	HPV Awareness	HPV Knowledge (Possible Range = 0–17)	OPC Awareness	OPC Knowledge (Range = 0–6)	HPV Vaccine Awareness	Vaccine Knowledge (Range = 0–5)
**1st Year** ***n* = 137**	87.6%	M = 9.91 SD = 3.66 *n* = 127	67.9%	M = 3.60 SD = 1.705 *n* = 97	80.3%	M = 2.20 SD = 1.232 *n* = 110
**2nd** ***n* = 232**	93.97%	M = 10.04 SD = 3.10 *n* = 221	70.26%	M = 3.47 SD = 1.467 *n* = 167	88.36%	M = 2.092 SD = 1.127 *n* = 205
**3rd** ***n* = 190**	92.93%	M = 10.46 SD = 3.514 *n* = 181	75.79%	M = 3.77 SD = 1.503 *n* = 145	90.00%	M = 2.5058 SD = 1.313 *n* = 172
**4th** ***n* = 224**	93.75%	M = 10.77 SD = 3.558 *n* = 214	83.48%	M = 3.86 SD = 1.3882 *n* = 190	87.50%	M = 2.4141 SD = 1.144 *n* = 198
**5th** ***n* = 48**	97.92%	M = 11.32 SD = 3.349 *n* = 47	89.58%	M = 3.98 SD = 1.4704 *n* = 44	95.83%	M = 2.543 SD = 1.5449 *n* = 46
**Graduate** ***n* = 167**	92.81%	M = 11.28 SD = 3.29 *n* = 157	85.63%	M = 3.61 SD = 1.46 *n* = 144	88.62%	M = 2.30 SD = 1.24 *n* = 148

**Table 3:** Awareness levels and mean (M), Standard Deviation (SD), and sample size (n) for knowledge scores by program of study.

Program of Study	HPV Awareness	HPV Knowledge Range = 0–17	OPC Awareness	OPC Knowledge Range = 0–6	Vaccine Awareness	Vaccine Knowledge Range = 0–5
**Arts &** **Humanities** ***n* = 45**	100%	M = 10.13 SD = 3.51 *n* = 45	68.89%	M = 3.69 SD = 1.71 *n* = 32	86.67%	M = 2.56 SD = 1.43 *n* = 39
**Business** ***n* = 82**	85.37%	M = 9.86 SD = 3.45 *n* = 71	65.85%	M = 3.35 SD = 1.58 *n* = 54	73.17%	M = 2.20 SD = 1.25 *n* = 60
**Education** ***n* = 1**	100%	12 correct	100%	3 correct	100%	5 correct
**Engineering** ***n* = 84**	88.10%	M = 9.80 SD = 3.66 *n* = 74	67.85%	M = 3.39 SD = 1.41 *n* = 59	87.80%	M = 2.28 SD = 1.36 *n* = 72
**Health Science** ***n* = 364**	96.15%	M = 11.29 SD = 3.20 *n* = 356	89.84%	M = 4.04 SD = 1.39 *n* = 328	94.23%	M = 2.43 SD = 1.18 *n* = 345
**Media Studies** ***n* = 60**	90.00%	M = 10.17 SD = 3.23 *n* = 59	66.67%	M = 8.57 SD = 1.53 *n* = 42	93.33%	M = 2.13 SD = 1.19 *n* = 56
**Law** ***n* = 37**	97.30%	M = 10.72 SD = 3.49 *n* = 36	67.57%	M = 3.44 SD = 1.39 *n* = 25	86.11%	M = 2.32 SD = 1.35 *n* = 31
**Music** ***n* = 55**	90.90%	M = 8.13 SD = 3.54 *n* = 54	70.90%	M = 2.95 SD = 1.50 *n* = 42	85.45%	M = 1.85 SD = 0.97 *n* = 48
**Medicine** ***n* = 18**	100%	M = 12.00 SD = 3.29 *n* = 18	77.79%	M = 4.43 SD = 1.34 *n* = 14	88.89%	M = 3.13 SD = 1.44 *n* = 16
**Science** ***n* = 123**	90.24%	M = 9.72 SD = 3.20 *n* = 114	72.36%	M = 3.65 SD = 1.54 *n* = 90	81.30%	M = 2.23 SD = 1.28 *n* = 100
**Social** **Science** ***n* = 135**	91.85%	M = 10.30 SD = 3.46 *n* = 126	75.56%	M = 3.53 SD = 1.48 *n* = 104	87.41%	M = 2.203 SD = 1.14 *n* = 118

Note: One participant was excluded from this summary because they did not indicate their program of study.

**Table 4:** Awareness levels and mean knowledge scores by ethnicity.

*Ethnicity*	HPV Awareness	HPV Knowledge (Range = 0–17)	OPC Awareness	OPC Knowledge (Range = 0–6)	Vaccine Awareness	Vaccine Knowledge (Range = 0–5)
**Caucasian** ***n* = 740**	94.73%	M = 10.79 SD = 3.44 *n* = 716	81.08%	M = 3.75 SD = 1.46 *n* = 608	91.76%	M = 2.35 SD = 1.22 *n* = 682
**All Other** **Ethnicities** ***n* = 265**	87.55%	M = 9.79 SD = 3.36 *n* = 238	67.55%	Mean = 3.50 SD = 1.58 *n* = 183	76.98%	M = 2.20 SD = 1.26 *n* = 204

**Table 5:** Awareness levels and mean (M), Standard Deviation (SD), and sample size (n) for knowledge scores by vaccination status.

*Vaccine Status*	HPV Awareness	HPC Knowledge (Range = 0–17)	OPC Awareness	OPC Knowledge (Range = 0–6)	Vaccine Awareness	Vaccine Knowledge (Range = 0–5)
**No Vaccine** ***n* = 434**	92.17%	M = 10.81 SD = 3.42 *n* = 406	77.42%	M = 3.69 SD = 1.46 *n* = 343	85.71%	M = 2.3431 SD = 1.25 *n* = 373
**Vaccine** ***n* = 412**	97.33%	M = 10.832 SD = 3.17 *n* = 411	82.77%	M = 3.909 SD = 1.43 *n* = 343	97.09%	M = 2.440 SD = 1.18 *n* = 402
**Unsure** ***n* = 154**	83.12%	M = 8.789 SD = 3.81 *n* = 133	62.99%	M = 2.89 SD = 1.55 *n* = 100	69.48%	M = 1.747 SD = 1.25 *n* = 107

Note: Five participants did not indicate their vaccine status and were therefore excluded.

**Table 6:** Correlational matrix for female knowledge scores.

	Age	Year of Study	HPV Knowledge	OPC Knowledge	Vaccine Knowledge	Total Knowledge
Age	1	0.836 **	0.156 **	0.169 **	0.070	0.179 **
Year of Study		1	0.158 **	0.219 **	0.090 *	0.201 **
HPV Knowledge			1	0.424 **	0.466 **	0.914 **
OPC Knowledge				1	0.339 **	0.707 **
Vaccine Knowledge					1	0.655 **
Total Knowledge						1

* *p* < 0.05, ** *p* < 0.01 level (2-tailed).

**Table 7:** Correlational matrix for male knowledge scores.

	Age	Year of Study	HPV Knowledge	OPC Knowledge	Vaccine Knowledge	Total Knowledge
Age	1	0.818 **	0.043	−0.045	0.037	0.024
Year of Study		1	0.071	−0.057	0.059	0.044
HPV Knowledge			1	0.444 **	0.556 **	0.937 **
OPC Knowledge				1	0.372 **	0.685 **
Vaccine Knowledge					1	0.710 **
Total Knowledge						1

** *p* < 0.01 level (2-tailed).

## Data Availability

These data are unavailable due to privacy or ethical restrictions.

## Supplementary Materials

The following supporting information can be downloaded at: https://www.mdpi.com/article/10.3390/cancers17030344/s1, File S1. Study Questionnaire.
