# Supplementary material for: An Assessment of Young Adults’ Awareness and Knowledge Related to the Human Papillomavirus (HPV), Oropharyngeal Cancer, and the HPV Vaccine
Source: Cancers (Basel). 2025 Jan 21;17(3):344. doi: 10.3390/cancers17030344 (PMC11816247; doi:10.3390/cancers17030344)
Supplement: Supplementary file 1 [file cancers-17-00344-s001.zip › cancers-3387136-supplementary.pdf]

<u>**Study Questionnaire**</u>

Demographics

(1) What is your age in years and the closest number of additional months (e.g., 20 years, 7 months)? Years, ______Months ______

(2) What gender do you identify with? Male Female Other

(3) What race/ethnicity do you identify with?

- Caucasian (white)
- Chinese
- South Asian (East Indian, Pakistani, Sri Lankan, etc.)
- Black
- Filipino
- Aboriginal
- Latin American
- Southeast Asian (Vietnamese, Cambodian, Malaysian, Laotian, etc.)
- Arab
- West Asian
- Korean
- Japanese
- Other (please specify) ______________________________

(4) Please identify your current level of education

- 1st year undergrad student
- 2nd year undergrad student
- 3rd year undergrad student
- 4th year undergrad student
- 5th year undergrad student
- Graduate Student
- Other (please specify) _________________________

(5) In which faculty are you enrolled?

- Arts and Humanities
- Business
- Dentistry
- Education
- Engineering
- Health Sciences
- Information and Media Studies
- Law
- Medicine and Dentistry
- Music
- Science
- Social Science

(6) Have you received one or more doses of the human papillomavirus (HPV) vaccination?

- Yes, one dose
- Yes, two doses
- Yes, three doses
- No

(7) Where did you obtain your knowledge of HPV?

- Family members
- School
- Friends
- A doctor or nurse
- The internet
- The media (television, radio, movies, etc.)
- Other (please specify) ________________________

HPV Awareness

(8) Prior to this survey, have you ever heard of the human papillomavirus (HPV)?[2]
Yes No

General Question on HPV

(9) On a scale of 1-5 (with 1 being 'not concerned at all' and 5 being 'extremely concerned', how concerned are you about becoming infected with HPV?[4]

Not concerned Extremely
at all concerned
1 2 3 4 5

(10) On a scale of 1-5 (with 1 being 'nothing' and 5 being 'very much/expert'), how much would you say you know about HPV?

Nothing A moderate amount Very much/Expert
1 2 3 4 5

HPV Knowledge

(11) HPV is a sexually transmitted infection[1,3] (T) True False I don't know
(12) HPV infection is rare in Canada[3] (F) True False I don't know
(13) A person could have HPV for many years without knowing it[3] (T)
True False I don't know
(14) Men cannot get HPV[1,3] (F) True False I don't know
(15) There are many different types of HPV[3] (T) True False I don't know
(16) HPV is the main cause of cervical cancer[1,2,3] (T) True False I don't know
(17) HPV infection can cause oropharyngeal (mouth and throat) cancer[2] (T) True
False I don't know
(18) HPV infection can cause genital warts[1,3] (T) True False I don't know
(19) HPV infection can cause HIV/AIDS[3] (F) True False I don't know
(20) HPV usually does not need any treatment[3] (T) True False I don't know
(21) HPV infection can cause anal cancer[2] (T) True False I don't know

HPV Risk Factors

(22) Having a higher number of sexual partners increases the risk of contracting HPV[2,3]
(T) True False I don't know
(23) One can become infected with HPV by having unprotected oral sex[1,2] (T)
True False I don't know
(24) Using condoms during intercourse completely protects one from becoming infected with HPV[1,2,3] (F) True False I don't know
(25) One can become infected with HPV by having unprotected anal sex[1,2] (T) True False I don't know
(26) Having sex at an earlier age increases the risk get getting HPV[3] (T) True
False I don't know

(27) Using an oral contraceptive (the birth control pill) protects me from becoming infected with HPV[2] (F) True False I don't know

Oropharyngeal Cancer Awareness

(28) Prior to this survey, have you ever heard of oropharyngeal cancers (cancer of the throat, base of tongue, soft palate, and/or the tonsils)?[2]

YES NO

General oropharyngeal cancer questions

(29) On a scale of 1-5 (with 1 being 'not concerned at all' and 5 being 'extremely concerned'), how concerned are you about getting oropharyngeal cancer?[4]

Not concerned Extremely
at all concerned
1 2 3 4 5

(30) On a scale of 1-5 (with 1 being 'nothing' and 5 being 'very much/expert'), how much would you say you know about oropharyngeal cancer?

Nothing A moderate amount Very much/Expert
1 2 3 4 5

Oropharyngeal Cancer Knowledge

(31) Both men and women can get oropharyngeal cancer[5] (T) True False I don't know
(32) The number of new cases of oropharyngeal cancer in Canada is increasing[6] (T) True False I don't know
(33) Smoking tobacco increases the risk for getting oropharyngeal cancer[5] (T) True False I don't know
(34) Having a high number of lifetime oral sex partners increases the risk for getting oropharyngeal cancer[5] (T) True False I don't know
(35) Drinking alcohol increases the risk of getting oropharyngeal cancer[5] (T) True False I don't know
(36) Having an HPV infection increases the risk of getting oropharyngeal cancer[2,5] (T) True False I don't know

HPV Vaccine Awareness

(37) Prior to this survey, have you ever heard of the HPV vaccine (brand names Gardasil© or Cervarix©)[2]

YES NO

HPV Vaccine Knowledge

(38) Men cannot obtain the HPV vaccine[7] (F) True False I don't know
(39) The HPV vaccine protects against cervical cancer[2,7] (T) True False I don't know
(40) The HPV vaccine protects against genital warts[7] (T) True False I don't know
(41) The HPV vaccine protects against oropharyngeal cancer[2] (T) True False I don't know
(42) Once vaccinated, women no longer have to receive regular cervical cancer screening (pap smears)[7] (F) True False I don't know

The items in the study questionnaire were based on information provided by, or adapted from, the following publications:

[1] Questions from: Ramirez et al. (1997) [97].

[2] Questions adapted from: Pelullo et al. (2012) [99].

[3] Questions adapted from: Bowyer et al. (2013) [100].

[4] Question adapted from: Gerend and Magloire (2008) [98].

[5] Questions were developed based on OPC risk factors identified by: Gillison et al. (2008) [102].

[6] Question was developed based on data from: Nichols et al. (2013) [53].
[7] Questions adapted from: Ragin et al. (2009) [74].
